# *Leishmania donovani* Internalizes into Host Cells *via* Caveolin-mediated Endocytosis

**DOI:** 10.1038/s41598-019-49007-1

**Published:** 2019-09-02

**Authors:** G. Aditya Kumar, Joyshree Karmakar, Chitra Mandal, Amitabha Chattopadhyay

**Affiliations:** 1 CSIR-Centre for Cellular and Molecular Biology, Uppal Road, Hyderabad, 500 007 India; 2 CSIR-Indian Institute of Chemical Biology, Raja S.C. Mullick Road, Kolkata, 700 032 India

**Keywords:** Mechanisms of disease, Phagocytosis

## Abstract

*Leishmania donovani* is an intracellular protozoan parasite that causes visceral leishmaniasis, a major cause of mortality and morbidity worldwide. The host plasma membrane serves as the portal of entry for *Leishmania* to gain access to the cellular interior. Although several host cell membrane receptors have been shown to be involved in the entry of *Leishmania donovani* into host cells, the endocytic pathway involved in the internalization of the parasite is not known. In this work, we explored the endocytic pathway involved in the entry of *Leishmania donovani* into host macrophages, utilizing specific inhibitors against two major pathways of internalization, *i.e*., clathrin- and caveolin-mediated endocytosis. We show that pitstop 2, an inhibitor for clathrin-mediated endocytosis, does not affect the entry of *Leishmania donovani* promastigotes into host macrophages. Interestingly, a significant reduction in internalization was observed upon treatment with genistein, an inhibitor for caveolin-mediated endocytosis. These results are supported by a similar trend in intracellular amastigote load within host macrophages. These results suggest that *Leishmania donovani* utilizes caveolin-mediated endocytosis to internalize into host cells. Our results provide novel insight into the mechanism of phagocytosis of *Leishmania donovani* into host cells and assume relevance in the development of novel therapeutics against leishmanial infection.

## Introduction

*Leishmania* is an obligate intracellular protozoan parasite that serves as the causative organism for leishmaniasis, a major cause of mortality and morbidity worldwide. The threat posed by leishmaniasis is significant particularly among economically weaker sections in tropical and subtropical regions^1–7^. Leishmaniasis accounts for ~1 million new cases and ~30,000 deaths annually^8^. Visceral leishmaniasis, one of the four types of the disease, is caused by *Leishmania donovani* and affects liver, spleen and bone marrow in humans^5^. The emergence of leishmaniasis as an opportunistic infection associated with HIV-1 infected patients^9,10^, and the growing drug resistance against the available drugs^11,12^ calls for greater impetus to develop novel therapeutic strategies against this disease.

*Leishmania* gains access to the host cell as a consequence of the bite of the infected female sandfly (*Phlebotomus spp*.) during its blood meal from a host^5,6,13^. In the course of its lifecycle, *Leishmania* exists in two distinct forms: (i) the flagellated extracellular promastigote form, and (ii) the amastigote form devoid of flagella that resides within host cells^5,6,13^. The first step for *Leishmania* toward establishing infection is to cross the plasma membrane of the host cell by phagocytosis^14–17^. The process of internalization has been shown to involve receptors on the host plasma membrane that recognize cognate ligands on the parasite^15,18,19^. These include the complement receptors CR1 and CR3, the fibronectin receptor, mannose-fucose receptor and receptor for advanced glycosylation end products.

An essential prerequisite for an intracellular pathogen to establish infection is to traverse the host plasma membrane^17,20–23^. Internalization of extracellular matter into the cellular interior is a complex, multistep process involving a concerted interplay between a large number of membrane-associated cellular components. In this context, we previously explored the role of host membrane lipids such as cholesterol and the underlying actin cytoskeleton in the entry and infection of *Leishmania donovani* using multiple complementary approaches^24–28^. The complexity involved in the process of internalization of pathogens is confounded by the multiplicity in pathways of phagocytosis exploited by the parasite. The major pathways implicated in internalization include clathrin- and caveolin-mediated endocytosis^29–31^. Although there appears to be an inherent redundancy in the membrane receptors implicated in the entry of different pathogens into host cells^32^, there is no general framework underlying the mechanism of phagocytosis of intracellular pathogens. For example, pathogenic bacteria such as *Listeria monocytogenes*, fungi such as *Candida albicans*, and viruses such as vesicular stomatitis virus have been shown to internalize into host cells in a clathrin-dependent manner^33,34^. On the other hand, caveolae have been implicated in the endocytosis of pathogens such as mycobacteria, *Plasmodium falciparum* and simian virus 40^35,36^.

Despite the wide variety of host cell membrane receptors implicated in the entry of *Leishmania donovani* into host cells, the endocytic pathway involved in this process has not been identified. In the present work, we have explored the cellular phagocytic machinery involved in the entry of *Leishmania donovani*. Using specific inhibitors of clathrin- and caveolin-mediated endocytosis, we show that *Leishmania donovani* utilizes caveolin-mediated phagocytosis to gain access to the host cell interior. Our results provide novel insight into the mechanism of phagocytosis of *Leishmania donovani* into host cells, and could assume relevance in the development of novel host-directed therapeutics against leishmanial infection that target the process of pathogen entry^37^.

## Results

### Inhibition of clathrin- and caveolin-mediated endocytosis in macrophages

Macrophages offer multiple pathways for the phagocytosis of intracellular pathogens. Clathrin- and caveolin-mediated endocytosis represent the most well studied and major pathways of internalization that have been implicated in the entry of several viral, bacterial, fungal and protozoan parasites^33–36^. However, as stated above, the endocytic machinery involved in the entry of *Leishmania donovani* has not been identified. To address this question, we studied the internalization of *Leishmania donovani* into macrophages upon treatment of host cells with specific inhibitors of clathrin- and caveolin-mediated endocytosis. Pitstop 2 inhibits clathrin-mediated endocytosis by associating with the terminal domain of clathrin, thereby obstructing the binding of clathrin to accessory proteins involved in orchestrating clathrin-mediated endocytosis^38^. Caveolin-mediated endocytosis could be inhibited using genistein, which is a tyrosine kinase inhibitor that blocks the phosphorylation of caveolin-1^39^.

**Figure 1:**
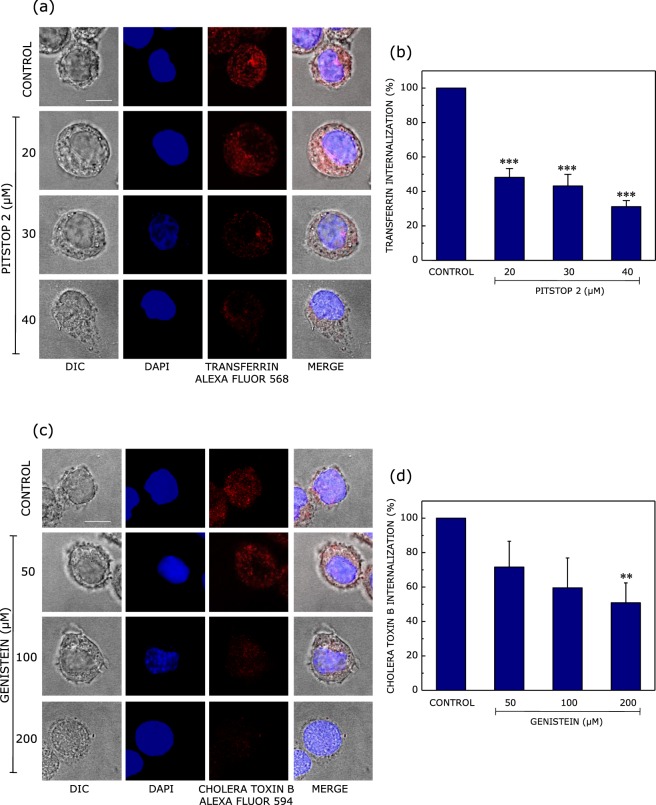
Pitstop 2 and genistein inhibit clathrin- and caveolin-mediated endocytosis, respectively, in macrophages. The effect of pitstop 2 and genistein on clathrin- and caveolin-mediated endocytosis was monitored by measuring the endocytosis of specific markers for each of the pathways of endocytosis. Panel (a) shows representative confocal microscopic images of the internalization of transferrin Alexa Fluor 568 (red) into J774A.1 macrophages (DIC images) upon treatment with increasing concentrations of pitstop 2. The macrophage nucleus is stained with DAPI (blue). Panel (b) shows the extent of internalization of transferrin quantified as the number of transferrin positive puncta per unit area, normalized to untreated cells. Data represent means ± S.E. of at least 5 independent measurements (***corresponds to significant (*p* < 0.001) difference in the internalization of transferrin into macrophages treated with pitstop 2 relative to control cells). Panel (c) shows representative confocal microscopic images of the internalization of cholera toxin B Alexa Fluor 594 into macrophages upon treatment with increasing concentrations of genistein. The extent of internalization of cholera toxin B quantified as cholera toxin B positive puncta per unit area, normalized to untreated cells, is shown in panel (d). Data represent means ± S.E. of at least 5 independent experiments (**corresponds to significant (*p* < 0.01) difference in the internalization of transferrin into macrophages treated with genistein relative to control cells). The scale bar represents 10 μm. See Materials and Methods for more details.

In order to evaluate the ability of pitstop 2 and genistein to inhibit clathrin- and caveolin-mediated endocytosis in macrophages, we monitored the internalization of known markers for these pathways upon treatment with increasing concentrations of inhibitors. Transferrin receptor has been shown to be a specific marker for clathrin-mediated endocytosis^40^. On the other hand, cholera toxin B has been shown to undergo internalization *via* caveolin-mediated endocytosis^41^. As shown in Fig. 1 (panels (a) and (b)), treatment of J774A.1 macrophages with increasing concentrations of pitstop 2 resulted in an inhibition of the endocytosis of transferrin, with ~69% reduction in transferrin internalization when macrophages were treated with 40 μM pitstop 2 (Fig. 1b). Similarly, Fig. 1 (panels (c) and (d)) show a reduction in the internalization of cholera toxin B upon treatment with genistein. We observed ~50% reduction in cholera toxin B internalization upon treatment with 200 μM genistein (Fig. 1d). Importantly, exposure of macrophages to pitstop 2 and genistein did not compromise cell viability under our experimental conditions (Fig. S1).

**Figure 2:**
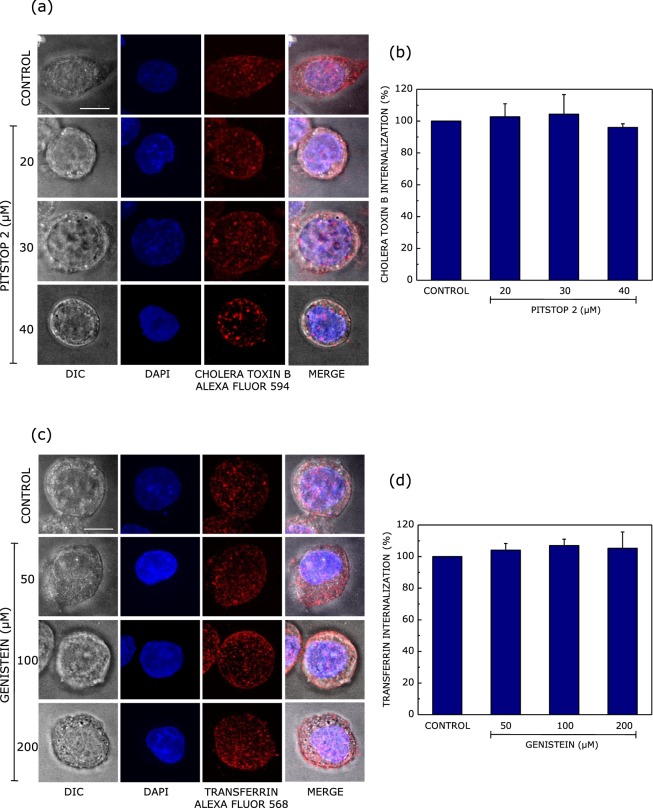
Pitstop 2 and genistein exhibit specificity toward inhibition of clathrin- and caveolin-mediated endocytosis. The specificity of the inhibitory effects of pitstop 2 and genistein were monitored by measuring the endocytosis of specific markers for caveolin- and clathrin-mediated endocytosis, respectively. Panel (a) shows representative confocal microscopic images of the internalization of cholera toxin B Alexa Fluor 594 (red) into macrophages (DIC images) upon treatment with increasing concentrations of pitstop 2. The macrophage nucleus is stained with DAPI (blue). The extent of internalization of cholera toxin B quantified as cholera toxin B positive puncta per unit area, normalized to untreated cells, is shown in panel (b). Data represent means ± S.E. of at least 5 independent experiments. Panel (c) shows representative confocal microscopic images of the internalization of transferrin Alexa Fluor 568 into macrophages upon treatment with increasing concentrations of genistein. Panel (d) shows the extent of internalization of transferrin quantified as the number of transferrin positive puncta per unit area, normalized to untreated cells. Data represent means ± S.E. of at least 5 independent measurements. The scale bar represents 10 μm. See Materials and Methods for more details.

In order to ensure that pitstop 2 and genistein exhibit *specificity* in their inhibitory effects on clathrin- and caveolin-mediated endocytosis, we tested the effect of these inhibitors on the internalization of cholera toxin B and transferrin, respectively. As shown in Fig. 2a, treatment of macrophages with increasing concentrations of pitstop 2 did not affect the internalization of cholera toxin B (specific marker for caveolin-mediated endocytosis). Similarly, the internalization of transferrin (specific marker for clathrin-mediated endocytosis) remained invariant when macrophages were treated with increasing concentrations of genistein (Fig. 2b). These results clearly indicate that pitstop 2 and genistein *specifically* inhibit clathrin- and caveolin-mediated endocytosis in J774A.1 macrophages, and could be used to explore the endocytic machinery involved in the internalization of *Leishmania donovani*.

### Pitstop 2 does not inhibit the entry of *Leishmania donovani* promastigotes into host macrophages

**Figure 3:**
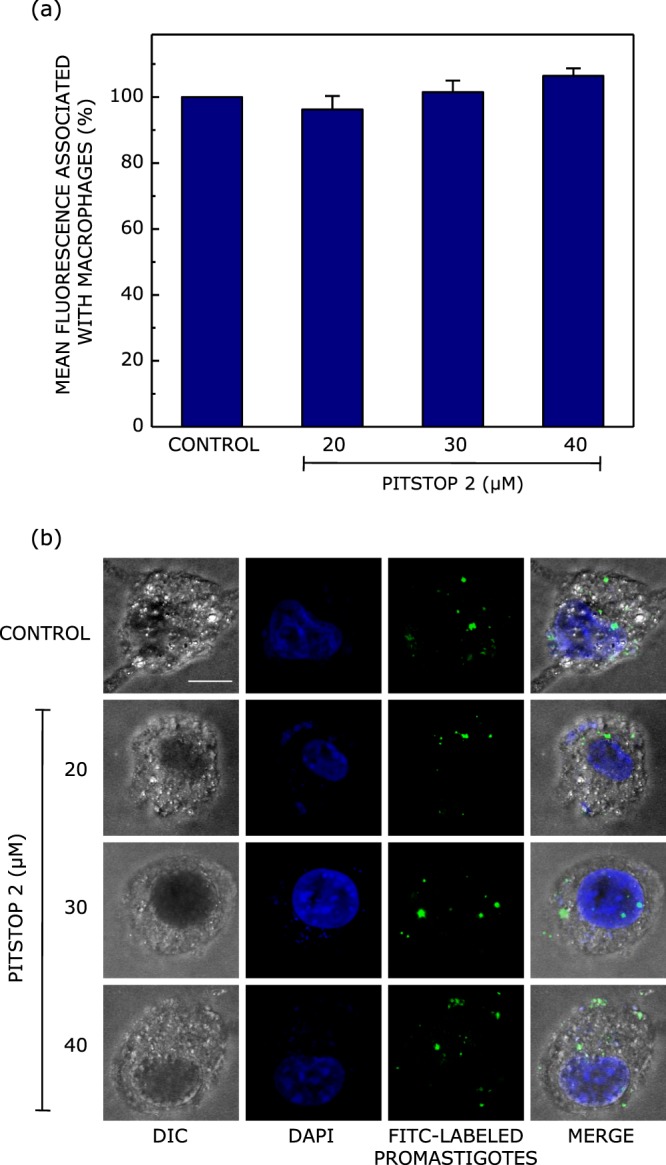
Inhibition of clathrin-mediated endocytosis does not affect internalization of *Leishmania donovani* into host macrophages. Macrophages were treated with increasing concentrations of pitstop 2 to inhibit clathrin-mediated endocytosis, and subsequently infected with FITC-labeled *Leishmania* promastigotes at a multiplicity of infection of 10:1 (parasite to macrophage). Fluorescence from membrane-bound (uninternalized) promastigotes was negated by treatment with trypan blue. Panel (a) shows quantitative flow cytometric estimates of internalized FITC-labeled promastigotes. Values are normalized to mean fluorescence associated with untreated (control) macrophages. Data represent means ± S.E. of at least four independent experiments. Panel (b) shows representative confocal microscopic images of FITC-labeled promastigotes (green) upon internalization into macrophages (DIC images) treated with increasing concentration of pitstop 2. Macrophage nuclei stained with DAPI are shown in blue. Merged images are shown in the panel on the extreme right. The scale bar represents 10 μm. See Materials and Methods for more details.

In order to delineate the endocytic pathway adopted by *Leishmania donovani* to enter into host macrophages, we inhibited clathrin- and caveolin-mediated endocytic routes using pitstop 2 and genistein, respectively, prior to infecting macrophages with *Leishmania donovani* promastigotes. We quantified the extent of phagocytosis of fluorescently (fluorescein isothiocyanate (FITC))-labeled *Leishmania* promastigotes into macrophages using flow cytometric analysis, complemented by confocal microscopic imaging. In order to account for only the internalized fraction of promastigotes, we quenched the fluorescence associated with membrane bound (uninternalized) promastigotes using trypan blue^42,43^ (see Materials and Methods for more details). We quantified the internalization of *Leishmania donovani* promastigotes into host macrophages upon treatment with the inhibitor of clathrin-mediated endocytosis, pitstop 2 (Fig. 3). As shown in Fig. 3a, internalization of *Leishmania donovani* promastigotes remains invariant upon treatment with increasing concentrations of pitstop 2. These observations were further confirmed by confocal microscopic imaging of macrophages infected with FITC-labeled *Leishmania* under these conditions (Fig. 3b). These results show that the internalization of *Leishmania donovani* into macrophages does not involve clathrin-mediated endocytosis.

### Treatment of host cells with genistein inhibits promastigote entry into host macrophages

**Figure 4:**
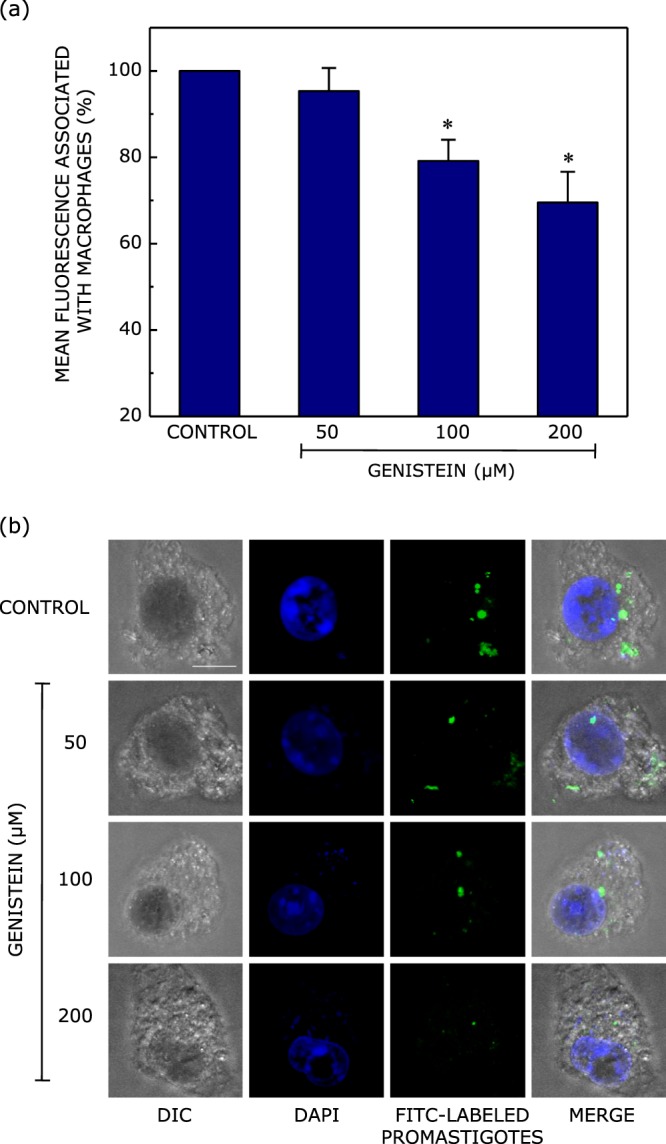
Inhibition of caveolin-mediated endocytosis leads to reduction in internalization of *Leishmania* promastigotes. Caveolin-mediated endocytosis in macrophages was inhibited by treating them with increasing concentrations of genistein. The macrophages were then infected with FITC-labeled *Leishmania* promastigotes at a multiplicity of infection of 10:1 (parasite to macrophage). Fluorescence from membrane-bound (uninternalized) promastigotes was negated by treatment with trypan blue. Quantitative flow cytometric estimates of internalized FITC-labeled promastigotes are shown in panel (a). Values are normalized to mean fluorescence associated with untreated (control) macrophages. Data represent means ± S.E. of at least three independent experiments (*corresponds to significant (*p* < 0.05) difference in mean fluorescence associated with macrophages treated with genistein relative to control cells). Panel (b) shows representative confocal microscopic images of FITC-labeled promastigotes (green) upon internalization into macrophages (DIC images) treated with increasing concentration of genistein. Macrophage nuclei were stained with DAPI (blue). The panel on the extreme right shows merged images. The scale bar represents 10 μm. See Materials and Methods for more details.

To assess the role of caveolin-mediated endocytosis in the entry of promastigotes into host cells, we treated macrophages with genistein prior to infection. Figure 4a shows a reduction in the entry of FITC-labeled promastigotes with increasing concentration of genistein. We observed ~30% reduction in the internalized promastigotes upon treatment with 200 μM genistein. As shown in Fig. 4b, confocal microscopic imaging of macrophages infected with FITC-labeled promastigotes reinforced our observation on the reduction in internalization of *Leishmania* promastigotes upon treatment with genistein. Taken together, these results show that *Leishmania donovani* promastigotes internalize into host cells *via* caveolin-mediated endocytosis.

### Intracellular amastigote load remains invariant upon inhibition of clathrin-mediated endocytosis

*Leishmania donovani* promastigotes, upon internalization into host cells, undergo morphological transformation into aflagellar amastigote forms. The amastigote forms of the parasite multiply within the host and spread the infection to other cells. In order to further explore the endocytic pathway for the entry of *Leishmania donovani* into macrophages, we measured the intracellular amastigote load within primary peritoneal macrophages upon treatment with inhibitors specific for clathrin- and caveolin-mediated endocytic pathways. The number of amastigotes within macrophages were visually scored after staining their nuclei with Giemsa.

**Figure 5:**
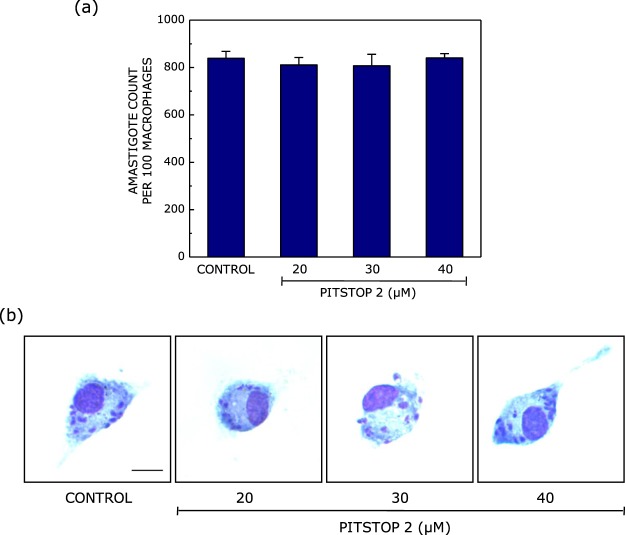
Inhibition of clathrin-mediated endocytosis does not affect the intracellular amastigote load. Peritoneal macrophages isolated from BALB/c mice were treated with increasing concentrations of pitstop 2 to inhibit clathrin-mediated endocytosis. Cells were infected with *Leishmania donovani* at a multiplicity of infection of 10:1 (parasite to macrophage). Panel (a) shows the number of intracellular amastigotes within macrophages estimated upon Giemsa staining. Data represent means ± S.E. of at least three independent experiments. Panel (b) shows representative microscopic images of *Leishmania*-infected macrophages stained with Giemsa. The larger nucleus seen in these images corresponds to the macrophage and the smaller nuclei correspond to intracellular amastigotes. The scale bar represents 10 μm. See Materials and Methods for more details.

We observed that treatment of macrophages with pitstop 2 did not affect the amastigote load within the host cells (Fig. 5a). Figure 5b shows representative microscopic images of pitstop 2 treated macrophages infected with *Leishmania*. These results are in agreement with the invariance observed in the entry of *Leishmania* promastigotes into host macrophages upon treatment with pitstop 2 (Fig. 3).

### Inhibition of caveolin-mediated endocytosis results in reduced amastigote load within host macrophages

**Figure 6:**
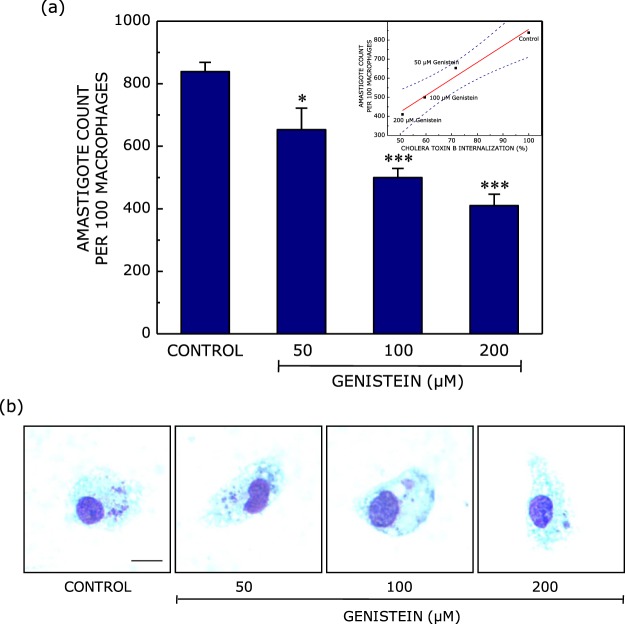
Reduction in intracellular amastigote load upon inhibition of caveolin-mediated endocytosis. Caveolin-mediated endocytosis in mouse peritoneal macrophages was inhibited upon treatment with increasing concentration of genistein. Macrophages were then infected with *Leishmania donovani* at a multiplicity of infection of 10:1 (parasite to macrophage). Panel (a) shows the number of intracellular amastigotes within macrophages estimated upon Giemsa staining. Data represent means ± S.E. of at least three independent experiments (*and ***correspond to significant (*p* < 0.05 and *p* < 0.001) difference in intracellular amastigote counts in macrophages treated with genistein relative to control cells). The inset shows correlation of the intracellular amastigote count (values taken from (**a**) and cholera toxin B internalization (from Fig. 1d) in macrophages with increasing concentrations of genistein. Linear regression analysis yielded a correlation coefficient (r) ~0.98. The significance of the correlation is apparent from the 95% confidence band (plotted as dashed lines). Panel (b) shows representative microscopic images of *Leishmania*-infected macrophages stained with Giemsa, where the larger nucleus corresponds to the macrophage and the smaller nuclei correspond to intracellular amastigotes. The scale bar represents 10 μm. See Materials and Methods for more details.

In order to assess the role of caveolin-mediated endocytosis in the internalization of *Leishmania donovani* into host cells, we measured the intracellular amastigote load within macrophages upon treatment with genistein. As shown in Fig. 6a, we observed a dose-dependent reduction in the intracellular amastigote load which reduced to ~50% when macrophages were treated with 200 μM genistein. Interestingly, the observed reduction in intracellular amastigote counts exhibited a tight correlation with the internalization of cholera toxin B (specific marker for caveolin-mediated endocytosis) with increasing concentrations of genistein (inset in Fig. 6a). Linear regression analysis between amastigote counts within macrophages (Fig. 6a) and cholera toxin B internalization (Fig. 1d) yielded a positive correlation coefficient (r) ~0.98, with all data points falling within the 95% confidence intervals, thereby reinforcing the role of caveolin-mediated endocytosis in the entry of *Leishmania donovani* into host cells. Representative microscopic images of genistein-treated macrophages infected with *Leishmania* amastigotes are shown in Fig. 6b. Taken together with our observation on reduction in promastigote entry upon treatment of host macrophages with genistein (Fig. 4), these results clearly suggest that *Leishmania donovani* phagocytose into host macrophages *via* caveolin-mediated endocytosis.

## Discussion

*Leishmania donovani* is an obligate intracellular protozoan parasite that enters into the host cell interior upon binding to host cell surface receptors such as the mannose-fucose receptor, receptor for advanced glycosylation end products, the fibronectin receptor, the Fc receptor and complement receptors CR1 and CR3^15,18,19^. However, the phagocytic mechanism involved in the process of internalization of *Leishmania donovani* into host cells is not yet explored. In this context, there appears to be no consensus in terms of the endocytic mechanism involved in the entry of pathogens into host cells. Both clathrin- and caveolin-mediated endocytic routes have been shown to be involved in the entry of various bacteria, virus, fungi and protozoan parasites^33–36^.

In order to address the endocytic pathway involved in the entry of *Leishmania donovani* into host cells, we quantified internalization of the parasite into host macrophages upon treatment of macrophages with specific inhibitors of clathrin- and caveolin-mediated endocytosis. Our results suggest that *Leishmania donovani* internalizes into host macrophages *via* caveolin-mediated endocytosis, since treatment with genistein (an inhibitor of caveolin-mediated endocytosis) resulted in reduction in the internalization of promastigotes as well as the intracellular amastigote load in host cells. Importantly, entry of *Leishmania donovani* promastigotes, as well as the intracellular amastigote load remained invariant upon treatment of host macrophages with pitstop 2, which specifically inhibits clathrin-mediated endocytosis. It should be noted that previous studies have implicated caveolae in the internalization of *Leishmania chagasi* into host cells^44,45^. Our present results extend these observations to *Leishmania donovani* by monitoring the role of both clathrin- and caveolin-mediated endocytic pathways in pathogen entry. It is noteworthy that several host plasma membrane receptors, such as the complement receptor CR3, that are involved in the process of internalization of *Leishmania donovani*, have been shown to be associated with caveolae and other proteins resident in caveolae such as GPI-anchored proteins^36,46,47^. These receptors on the host plasma membrane have been suggested to bind to cognate ligands such as the metalloprotease glycoprotein 63 (GP63) and lipophosphoglycan (LPG) on the pathogen membrane as the first step toward internalization of *Leishmania* into host macrophages^15,16^.

Caveolae have been suggested to be plasma membrane-associated portals enriched in cholesterol and are implicated in essential cellular processes such as signal transduction and endocytosis^48,49^. In this context, caveolin-1, the major structural protein constituting caveolae, has been shown to specifically bind cholesterol *via* sequence motifs known as cholesterol recognition/interaction amino acid consensus (CRAC) motif^50,51^. Interestingly, using several complementary approaches, we have previously highlighted the role of host membrane cholesterol in the entry of *Leishmania donovani* into host macrophages^24–26,28^. In addition, we observed the presence of multiple CRAC and CARC (inverted CRAC) motifs in transmembrane domains of receptors implicated in pathogen entry^17^. Our present results on the involvement of caveolae as portals for the entry of *Leishmania donovani* reinforce our previous observations on the host membrane cholesterol-dependence of parasite entry.

Taken together, our results show that *Leishmania donovani* internalizes into host macrophages *via* caveolin-mediated endocytosis. These results are significant in delineating the mechanistic basis of internalization of *Leishmania* into host cells. In addition, our results could have implications in the development of therapeutic strategies that target the host endocytic machinery, which could help overcome the challenges involved in drug resistance.

## Materials and Methods

### Materials

Antibiotic antimycotic solution, genistein, gentamicin sulfate, IMDM (Iscove’s Modified Dulbecco’s Medium), M-199 (Medium-199), MTT (3-(4,5-dimethylthiazol-2-yl)-2,5-diphenyl-tetrazolium bromide), FITC (fluorescein isothiocyanate), trypan blue and Giemsa stain were obtained from Sigma Chemical Co. (St. Louis, MO). Fetal calf serum (FCS) was from Gibco/Life Technologies (Grand Island, NY). Pitstop 2 was obtained from Abcam (Cambridge, MA). Transferrin Alexa Fluor 568 conjugated and cholera toxin B Alexa Fluor 594 conjugated were purchased from Molecular Probes/Invitrogen (Eugene, OR). All other chemicals and solvents used were of the highest available purity. Water was purified through a Millipore (Bedford, MA) Milli-Q system and used throughout.

### Cell culture

J774A.1 murine macrophages (American Type Culture Collection) were cultured as described previously^28^. See Supplementary Information (section S1) for more details.

### Parasite culture

*Leishmania donovani* strain AG83 (MHOM/IN/1983/AG83) promastigotes were maintained as described previously^28^. See Supplementary Information (section S2) for more details.

### Isolation of murine primary peritoneal macrophages

Peritoneal macrophages were isolated from BALB/c mice as described earlier^28^. See Supplementary Information (section S3) for more details.

All animal-related experiments were carried out in accordance with the National Regulatory Guidelines issued by Committee for the Purpose of Control and Supervision of Experiments on Animals (CPCSEA), Ministry of Environment and Forest (Govt. of India). Use of BALB/c mice was approved by the Institutional Animal Ethics Committee of CSIR-Indian Institute of Chemical Biology, Kolkata, India with license number 147/1999/CPCSEA. BALB/c mice were housed under standard condition of temperature (25 ± 1 °C), relative humidity (55 ± 10%) and 12 h/12 h light/dark cycles and fed with standard diet.

### Inhibition of clathrin- and caveolin-mediated endocytosis

Pitstop 2 was used to inhibit clathrin-mediated endocytosis in macrophages. A 20 mM stock solution of pitstop 2 was prepared in dimethyl sulfoxide (DMSO) and cells were treated with 20, 30 and 40 μM of pitstop 2 in serum-free medium at 37 °C for 30 min. Caveolin-mediated endocytosis was inhibited using genistein. A stock solution of 200 mM genistein was prepared in DMSO and cells were treated with 50, 100 and 200 μM genistein in serum-free medium at 37 °C for 30 min. Cells were washed twice with PBS after the treatment. It was ensured that the amount of DMSO was always <0.2% (v/v), such that treatment of cells with similar amounts of DMSO did not affect the cellular morphology.

### MTT cell viability assay

MTT assay was performed to test the viability of cells upon treatment with pitstop 2 and genistein, as described earlier^28,52^. See Supplementary Information (section S4) for more details.

### Quantitation of internalization of transferrin and cholera toxin B

Macrophages were incubated with 10 μg/ml transferrin Alexa Fluor 568 conjugate (or cholera toxin B Alexa Fluor 594 conjugate) in serum-free medium at 37 °C for 30 min. Cells were transferred to ice and washed twice with cold PBS to remove excess unbound transferrin (or cholera toxin B). Cell membrane bound transferrin (or cholera toxin B) was removed by incubating with 50 mM acetic acid, 150 mM NaCl solution for 15 min. Cells were then fixed with 4% (v/v) formaldehyde, washed with PBS and mounted in media containing DAPI. Microscopy was performed on a Leica SP8 confocal microscope (Wetzlar, Germany). Transferrin Alexa Fluor 568 conjugate was excited at 561 nm and emission was collected between 570–640 nm. Cholera toxin B Alexa Fluor 594 conjugate was excited at 594 nm and emission was collected between 600–640 nm. Z-section images with a fixed step size of 0.5 μm were acquired using a 63x/1.4 NA oil immersion objective under 1 airy condition. Internalized transferrin Alexa Fluor 568 conjugate (or cholera toxin B Alexa Fluor 594 conjugate) was quantified using ImageJ (National Institutes of Health, Bethesda) by counting their respective puncta normalized to area enclosed by the maximum intensity projection generated by merging all z-sections.

### Flow cytometric analysis of internalized promastigotes

Flow cytometric analysis of internalized *Leishmania donovani* was carried out as described previously^28^ with some modifications. See Supplementary Information (section S5) for more details.

### Confocal microscopic imaging of promastigotes

Confocal microscopic imaging of macrophages infected with FITC-labeled *Leishmania donovani* promastigotes was carried out as described previously^28^ with some modifications. See Supplementary Information (section S6) for more details.

### Quantitation of intracellular amastigote load

The intracellular amastigote load within peritoneal macrophages derived from BALB/c mice was estimated as described previously^28^ with some modifications. See Supplementary Information (section S7) for more details.

### Statistical analysis

Student’s two-tailed unpaired *t*-test was performed using GraphPad Prism software version 4.0 (San Diego, CA) to analyze significance levels. The correlation between intracellular amastigote load and the internalization of cholera toxin B into macrophages treated with increasing concentrations of genistein was analyzed using the same software. Plots were generated using OriginPro version 8.0 (OriginLab, Northampton, MA).

## Supplementary information

Supplementary Information
