# Supplementary material for: Leishmania donovani Internalizes into Host Cells via Caveolin-mediated Endocytosis
Source: Sci Rep. 2019 Sep 2;9:12636. doi: 10.1038/s41598-019-49007-1 (PMC6718660; doi:10.1038/s41598-019-49007-1)
Supplement: Supplementary file 1 — Supplementary Information [file 41598_2019_49007_MOESM1_ESM.pdf]

Supplementary Information

Leishmania donovani Internalizes into Host Cells *via* Caveolin-mediated Endocytosis

G. Aditya Kumar[a,†], Joyshree Karmakar[b,†],
Chitra Mandal[b,*], and Amitabha Chattopadhyay[a,*]

[†]Equal contribution

[a]CSIR-Centre for Cellular and Molecular Biology, Uppal Road,
Hyderabad 500 007, India; [b]CSIR-Indian Institute of Chemical Biology,
Raja S.C. Mullick Road, Kolkata 700 032, India

*Address correspondence to Amitabha Chattopadhyay, Tel: +91-40-2719-2578, Fax: +91-40-2716-0311, E-mail: amit@ccmb.res.in; or Chitra Mandal, Tel: +91-33-2429-8861, Fax: +91-33-2473-5197, E-mail: cmandal@iicb.res.in

S1. Cell culture. Cells were maintained in IMDM medium supplemented with 2.4 g/l sodium bicarbonate, 10% heat-inactivated FCS, and antibiotic antimycotic (100 U/ml penicillin, 100 μg/ml streptomycin, and 0.25 μg/ml amphotericin B) solution in a humidified atmosphere with 5% CO_2 at 37 °C.

S2. Parasite culture. *Leishmania* promastigotes were maintained in M-199 medium supplemented with 200 μg/ml gentamicin sulfate and 10% heat-inactivated FCS at 22 °C. The virulence property of promastigotes was routinely checked by testing their capacity to infect hamsters.

S3. Isolation of murine primary peritoneal macrophages. Chilled PBS (10 ml) was injected into the peritoneal cavity of 8-10 week old BALB/c mice. Peritoneal exudates collected in chilled PBS were centrifuged and washed with PBS. Cells were subsequently suspended in IMDM medium containing 10% heat-inactivated FCS and plated on glass coverslips at a density of ~$5x10^4$. Cells were incubated at 37 °C in a humidified atmosphere with 5% CO_2. Cover slips were washed after 24 h with PBS to remove non-adherent cells, and adhered macrophages were incubated for 24 h prior to further experiments.

S4. MTT cell viability assay. Macrophages in the mid log phase were plated at a density of ~$1x10^4$ in 96-well plates and treated with different concentrations of pitstop 2 or genistein. MTT was dissolved in serum-free IMDM medium and added to macrophages at a final concentration of 0.5 mg/ml, followed by incubation at 37 °C for 3 h. Formazan crystals formed upon reduction of MTT salt by mitochondrial enzymes in live cells[1] are insoluble in aqueous medium and the formazan crystals formed were subsequently dissolved in DMSO after discarding the medium. The color obtained was measured by absorbance at 550 nm using an EnSpire 2300 multimode reader (Perkin Elmer, MA).

S5. Flow cytometric analysis of internalized promastigotes. Promastigotes in the mid log phase were incubated with 0.1% FITC in 50 mM carbonate buffer, pH 8.0, at room

tempearuture (~23 °C) for 1 h. Unbound FITC was thoroughly washed off using PBS and the parasites were then suspended in IMDM medium containing 2% heat-inactivated FCS. Flow cytometry was used to confirm uniform labeling of parasites with FITC. J774A.1 macrophages were infected with FITC-labeled promastigotes at 37 °C for 1 h at a multiplicity of infection of 10:1 (parasite to macrophage). Subsequent to infection, cells were washed and incubated with 0.02% trypan blue in PBS for 5 min to quench fluorescence associated with membrane bound (uninternalized) promastigotes. This method allows selective quantitation of only the internalized population of FITC-labeled promastigotes.[2,3] Macrophages were washed and suspended in PBS for flow cytometric analysis. Flow cytometry was performed on a FACS Calibur flow cytometer (BD Biosciences, San Jose, CA), and the mean fluorescence of internalized FITC-labeled promastigotes associated with 10,000 macrophages was measured and analyzed using the CellQuest Pro analysis software.

S6. Confocal microscopic imaging of promastigotes. J774A.1 macrophages were plated at a density of ~2x10^4 on glass coverslips and grown in IMDM medium for 24 h. Subsequent to treatment with inhibitors of endocytosis, cells were infected with FITC-labeled promastigotes for 1 h. Cells were washed and incubated in 0.02% trypan blue in PBS for 5 min to quench the fluorescence associated with uninternalizaed membrane bound promastigotes. Cells were then washed with PBS, fixed with 4% (v/v) formaldehyde, and mounted in media containing DAPI. Z-section images were acquired on a Leica SP8 confocal microscope (Wetzlar, Germany) with a 63x/1.4 NA oil immersion objective, and maximum intensity projections were generated upon merging the z-sections.

S7. Quantitation of intracellular amastigote load. Peritoneal macrophages derived from BALB/c mice were grown on glass coverslips and infected with *Leishmania* promastigotes at a multiplicity of infection of 10:1 (parasite to macrophage) for 1 h at 37 °C. Cells were then washed to remove unbound parasites and further incubated for 20 h under similar conditions. Coverslips were subsequently washed with PBS, air dried, fixed with methanol and stained with Giemsa. Cells were imaged on an Evos XL Cell Imaging System (Life Technologies,

CA) microscope using a 100x oil immersion objective. Giemsa-stained intracellular amastigotes were scored visually, and normalized to 100 macrophages.

References

1. Vistica, D. T., Skehan, P., Scudiero, D., Monks, A., Pittman, A. & Boyd, M. R. Tetrazolium-based assays for cellular viability: a critical examination of selected parameters affecting formazan production. *Cancer Res.* **51**, 2515-2520 (1991).
2. Loike, J. D. & Silverstein, S. C. Fluorescence quenching technique using trypan blue to differentiate between attached and ingested glutaraldehyde-fixed red blood cells in phagocytosing murine macrophages. *J. Immunol. Methods* **57**, 373-379 (1983).
3. Roy, S. & Mandal, C. *Leishmania donovani* utilize sialic acids for binding and phagocytosis in the macrophages through selective utilization of siglecs and impair the innate immune arm. *PLoS Negl. Trop. Dis.* **10**, e0004904 (2016).

Figure S1
Kumar *et al.*

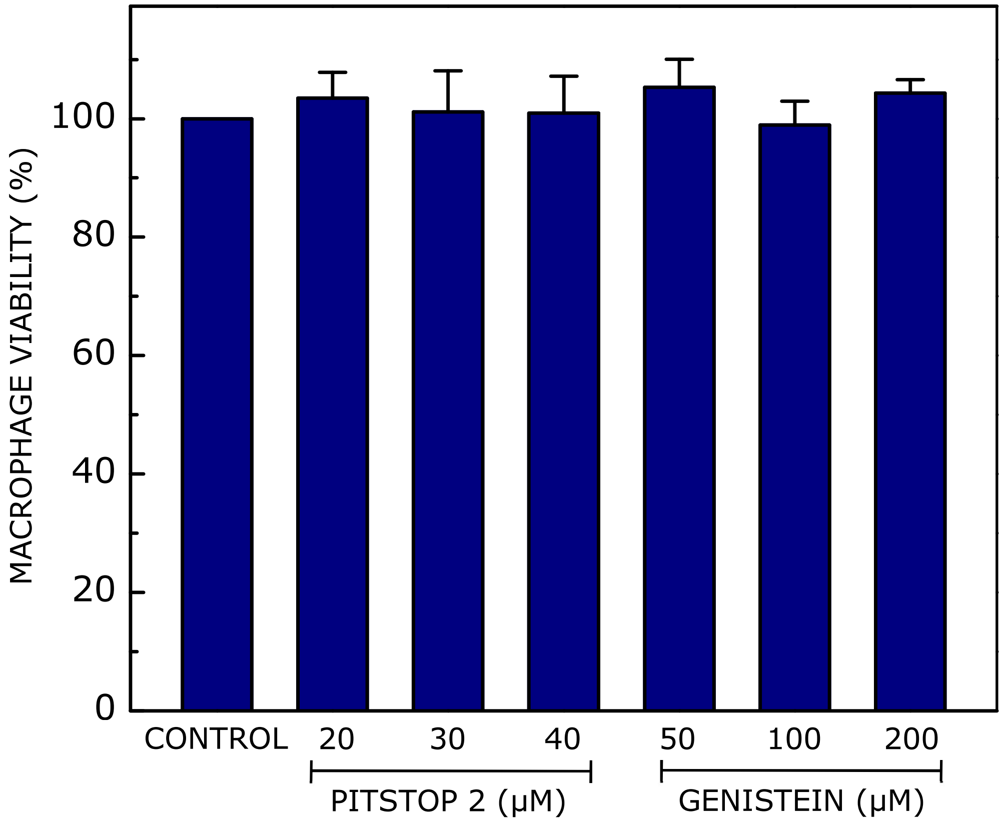

Fig. S1. Effect of endocytosis inhibitors on macrophage viability. J774A.1 cells were tested for viability using MTT assay upon treatment with increasing concentrations of pitstop 2 and genistein. Values are expressed as percentages of viability normalized to untreated (control) cells. Data represent means ± S.E. of at least 6 independent experiments. See Materials and Methods for other details.
